# Adiposity, metabolites and endometrial cancer risk: inference from combinations of Mendelian randomization and observational analyses

**DOI:** 10.1186/s12885-025-14756-y

**Published:** 2025-10-21

**Authors:** Matthew A. Lee, Vanessa Y. Tan, Dimitri J. Pournaras, Sabrina Wang, Laure Dossus, Marc J. Gunter, Kaitlin H. Wade, Laura J. Corbin, Nicholas J. Timpson

**Affiliations:** 1 Medical Research Council (MRC) Integrative Epidemiology Unit, Population Health Sciences, Bristol Medical School, University of Bristol, Oakfield House, Oakfield Grove, Bristol, BS8 2BN UK; 2 Population Health Sciences, Bristol Medical School, University of Bristol, Bristol, UK; 3 International Agency for Research on Cancer, World Health Organization, Lyon, France; 4 Department of Upper GI and Bariatric/Metabolic Surgery, North Bristol NHS Trust, Southmead Hospital, Bristol, UK; 5 Cancer Epidemiology and Prevention Research Unit, School of Public Health, Imperial College London, London, UK

**Keywords:** Adiposity, Metabolites, Endometrial cancer, Mendelian randomization

## Abstract

**Introduction:**

The associations between excess adiposity and endometrial cancer (EC) risk may be mediated by altered metabolic profiles. Here, we triangulated evidence from observational and Mendelian randomisation (MR) analyses to investigate the relationship between adiposity traits, circulating metabolites, and their effects on endometrial cancer.

**Methods:**

Observational analyses were performed in UK Biobank (N cases and controls = 1,005 and 215,339, respectively). Univariable and multivariable MR analyses were performed using female-specific summary statistics for adiposity traits (GIANT consortium; N BMI and WHR = 434,793 and 281,153, respectively), circulating metabolites (UK Biobank; *N* = 140,768) and EC (Endometrial Cancer Association Consortium; N cases and controls = 12,906 and 108,979, respectively).

**Results:**

Higher body mass index (BMI) was associated with increased odds of overall EC, endometrioid EC, and non-endometrioid EC in both observational and MR analyses; however, there was weaker evidence for waist-hip-ratio (WHR). BMI was associated with 165 metabolites, 25 of which were associated with EC risk. Multivariable MR analyses suggest that several lipid metabolites and ratios may mediate the association between BMI and non-endometrioid EC, although analyses using Phenoscanner suggest that alternative pathways such as height and blood cell traits could influence the EC risk.

**Conclusion:**

Evidence here suggests that higher BMI causes a higher risk of overall and all histological subtypes of EC and variation in numerous circulating metabolites. Several of these metabolites showed relationships consistent with an intermediate role between BMI and non-endometrioid EC, however, further analyses highlighted other potential shared mechanisms that could influence the risk of EC.

**Supplementary Information:**

The online version contains supplementary material available at 10.1186/s12885-025-14756-y.

## Introduction

Endometrial cancer (EC) is the most common gynaecological cancer among women [1]. Based on differences in histology and clinical outcomes, there are two main subtypes: endometrioid carcinomas with good prognosis and non-endometrioid carcinomas with worse prognosis [2]. Endometrioid EC is more commonly hormonally driven compared with non-endometrioid EC [3].

Excess adiposity is associated with EC in observational studies [4]. Mendelian randomisation [5] (MR) studies, which use genetic variants as instruments (or proxies), of adiposity traits further support the evidence base for a causal relationship with EC [6–8]. However, the underlying mechanisms involved in the adiposity-EC relationship remains incomplete. Furthermore, evidence of associations between body fat distribution, measured as waist-hip-ratio (WHR), has only been supported with observational studies [4, 6, 8].

Metabolic reprogramming is recognised as a hallmark of tumorigenesis [9] and there is evidence that metabolic dysfunction drives the development and progression of EC [10, 11]. Findings from a recent prospective study suggest that concentrations of glycine, serine, sphingomyelin and free carnitine may drive EC development [12]. Increased adiposity causes changes to an individual’s systemic metabolic profile [13–15]. Observational and MR studies support an effect of adiposity (proxied using body mass index [BMI]), on raised amino acids, fatty acids and inflammatory glycoprotein acetyls [15], leading to suggestions that a potential mechanism linking adiposity and EC could be adiposity-induced metabolic changes [10]. A recent prospective study showed that EC is positively associated with adiposity-associated metabolic changes including specific amino acids and lipids [16]; however, whether these relationships are causal is unclear.

We aimed to better understand the potential role of circulating metabolites as intermediates in the association between adiposity and EC risk by triangulating evidence from univariable two-step [17] MR and multivariable MR approaches in combination with observational analyses in UK Biobank.

## Methods

### Analytical strategy

**Fig. 1:**
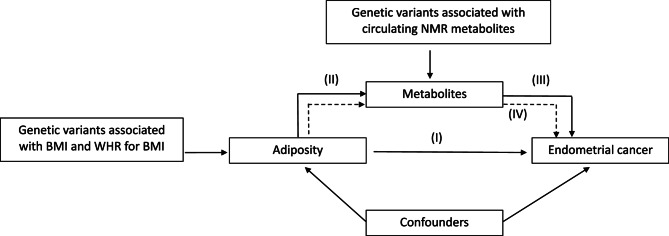
Study overview

This study has four main analyses that were performed sequentially (Fig. 1) to estimate: (Part I) the effect of adiposity measures on EC, (Part II) the effect of adiposity measures on circulating metabolites, (Part III) the effect of adiposity-associated metabolites on EC, and (Part IV) the potential intermediate role of adiposity-associated metabolites in the relationship between adiposity and EC (identified in Part II and III). Observational analyses were performed for Parts I-III. MR analyses were performed for Parts I-IV; Metabolite data from UK Biobank were used in both the MR and observational analyses. This study is reported as per the Strengthening the Reporting of Observational Studies in Epidemiology (STROBE) and STROBE-MR guidelines (Supplementary Table S1 and S2) [18, 19].

### Observational analyses (Part I-III)

#### Study population

UK Biobank is a biomedical database of more than 500,000 participants (aged 37–73 years, 56.3% were women) recruited between 2006 and 2020 [20, 21]. In addition to the collection of biological samples (blood, saliva and urine), health, demographic and anthropometric data were collected in 22 assessment facilities across England, Wales and Scotland [22]. Participants provided written informed consent. Ethical approval was obtained from the Northwest Multi-centre Research Ethics Committee (11/NW/0382).

#### Adiposity measures

BMI was calculated as weight (kg)/height^2^ (m^2^) and WHR as waist circumference (cm)/hip circumference (cm). Height was measured to the nearest centimetre, using a Seca 202 stadiometer, and body weight to the nearest 0.1 kg, using a Tanita BC-418 body composition analyser. Waist circumference was measured at the natural indent (the umbilicus was used if the natural indent could not be observed); hip circumference was measured at the widest part of the hips. BMI and WHR were inverse rank normal transformed prior to analyses and represent normalised SD units.

#### Metabolite measures

A random subset of non-fasting plasma samples, consisting of 275,000 participants and 17,000 repeat-visit samples (~ 15,500 of these have both a baseline and repeat assessment), were quantified using ^1^H-Nuclear magnetic resonance (^1^H-NMR) metabolomics (Nightingale Health Ltd; biomarker quantification version 2020 [23, 24]; data pre-processing and quality control steps are described previously [24, 25]). A total of 249 metabolic traits (168 concentrations plus 81 ratios) were quantified. Metabolite concentrations were inverse ranked normal transformed prior to analyses and represent normalised SD units.

#### Endometrial cancer

The UK Biobank database contained a record of all cancers including their subtype occurring either before or after participant enrolment using the International Classification of Diseases, 9th and 10th revision (ICD-10, ICD-9). The following ICD-10 and ICD-9 codes were used to define EC: ICD10 codes (C540, C541, C542, C543, C549 and C55) and ICD9 codes (179, 1799, 180, 182, 1820, 1821 and 1828). Cases were characterised as incident or prevalent using ‘age when they attended the centre’ and ‘age when first reported EC cancer’. Participants were defined as incident cases if their ‘attending age’ was less than their ‘cancer diagnosis age’. In total, there were 1,935 EC cases with 1,005 being incident cases and 930 prevalent cases. Only incident cases (*N* = 1,005) were included in our analyses. Controls (*n* = 215,339) were defined as female participants who had no record of any type of cancer, in-situ carcinoma, or an undefined neoplasm.

#### Covariables

We included the following potential confounders of the BMI-metabolite-EC relationships: age at assessment, physical activity, smoking status, alcohol consumption and educational attainment) and history of hormone replacement therapy (HRT) use, age at first live birth, age at last live birth, age at menarche and menopausal status. Age, smoking status, alcohol consumption, education attainment and female-specific factors were self-reported at the baseline assessment by questionnaire. Physical activity level over a typical week was self-reported using the International Physical Activity Questionnaire and reported as metabolic equivalent of task (MET) per week.

#### Statistical analyses

Observational associations between: (I) adiposity measures and EC (Fig. 1, Part I) were assessed using multivariable logistic regression, (II) adiposity measures and metabolites (Fig. 1, Part II) were assessed using multivariable linear regression for cases and controls combined in one cohort and (III) adiposity-associated metabolites and EC (Fig. 1, Part III) were assessed using multivariable logistic regression. All analyses were initially adjusted for age at assessment and centre. Models were additionally adjusted for potential confounders.

For our hypothesis-free discovery analysis examining the association between BMI and circulating metabolites, we applied a Bonferroni-adjusted *p*-value threshold (0.05/249) to account for multiple testing. In a subsequent hypothesis-driven analysis, we investigated the relationship between BMI-associated metabolites (identified in the discovery analysis) and endometrial cancer outcomes. For these analyses, we used a *p*-value < 0.05 and did not apply a multiple testing correction.

### Genome-wide association study results and Mendelian randomisation analyses (Part I-IV)

#### Data sources and study populations

##### Adiposity instruments

We identified single nucleotide polymorphisms (SNPs) that were independently associated (linkage disequilibrium (LD), R^2^ < 0.001) with BMI and WHR at *p* < 5 × 10^−9^ from a recent large-scale female-specific genome-wide association study (GWAS) meta-analysis of 434,794 individuals of European ancestries from the Genetic Investigation of Anthropometric Traits (GIANT) consortium and the UK Biobank [26] (Supplementary Table 3). Adiposity measures were inverse rank normal transformed prior to genome-wide analysis and represent a normalised standard deviation (SD). In total, 271 (average F-statistic = 41) and 227 (average F-statistic = 45) SNPs were identified for BMI and WHR, respectively.

##### Metabolite GWAS in UK Biobank and selection of metabolite instruments

We conducted a female-specific GWAS of the 249 ^1^H-NMR-derived metabolites and ratios in UK Biobank female participants of European descent (*N* = 140,768) using the MRC IEU UK Biobank GWAS pipeline [27] (Supplementary methods). Genome-wide association analysis was conducted using a linear mixed model (LMM) as implemented in BOLT-LMM (v2.3) [28]. We identified 11–167 independent SNPs (r^2^ < 0.001 and *p* < 5 × 10^−8^) for each of the ^1^H-NMR-derived metabolites (average F-statistics: ranged from 40.6 to 81.6).

##### Endometrial Cancer GWAS data

We obtained SNP estimates from the largest GWAS (as log odds ratio (OR)) for EC to date [8], including up to 12,906 cases and 108,979 controls from 13 studies (Supplementary Table 3). Of the 12,906 cases and 108,979 controls, 636 cases (5%) and 62,853 controls (58%) were from UK Biobank. Summary statistics were also available for the association between genetic variation and the EC subtypes, including 8,758 endometrioid and 1,230 non-endometrioid cases; both GWASs used the full set of controls (*N* = 108,979). None of the endometrial subtype cases were from UK Biobank. Histological subtypes of EC were confirmed based on pathology reports and detailed study descriptions have previously been reported [8].

#### Statistical analysis

MR estimates are interpreted as the change in outcome per SD unit change in exposure. Estimates for metabolite outcomes reflect SD unit change, and estimates for EC outcomes reflect odds ratios (OR).

Details of the SNPs included in each analysis, and proxies used (where SNPs were not available in the outcome data), are provided in Supplementary Table 4. Univariable causal estimates were combined using the inverse-variance weighted (IVW) multiplicative random effects (IVW-MRE) model [29]. Where possible (i.e., where there were three or more instruments), the assumption of no pleiotropy among genetic instruments and outcomes were explored using MR-Egger [30], weighted median [31] and weighted mode [32] estimators. These methods are sensitive to the effects of potential pleiotropy under different assumptions. No *p*-value threshold requirements were set for these methods and, instead, consistency between the IVW model and the three sensitivity MR methods (MR-Egger, weighted median and weighted mode) was assessed.

Adiposity traits with an effect on EC with *p* values < 0.05 from IVW-MRE models and with consistent associations across the three sensitivity MR methods, were taken forward and examined for associations with the ^1^H-NMR-measured metabolites using the same four models (Fig. 1, Part II). Metabolites that were associated with the adiposity traits with *p* values < 0.05/249 (Bonferroni-adjusted *p*-value threshold) from IVW-MRE models were taken forward and examined for association with EC risk using IVW-MRE model (if $\:\ge\:$2) or Wald ratio (if 1 SNP) (Fig. 1, Part III).

Multivariable MR (MVMR) [33, 34] was conducted to test the hypothesis that adiposity-associated metabolites (identified in Part IIand III) may act as intermediates in the relationship between BMI and EC (Fig. 1, Part IV). Metabolites that showed a consistent direction of effect across the adiposity-EC, adiposity-metabolite, and metabolite-EC analyses from both MR and observational analyses (Fig. 1, Part II and III) were included in the MVMR analyses. For the MVMR analyses, we included each BMI-associated and EC-associated metabolite in turn to estimate the direct causal effect of BMI on EC. We used female-specific SNPs for BMI based on an earlier (lower-powered) GWAS [35] for the multivariable models to avoid a relative dilution of metabolite instrument strength [36] given that the number of SNPs for BMI from the latest BMI GWAS far outnumbered those for metabolites. Conditional F-statistics were used to evaluate instrument strength [34]. Heterogeneity was quantified using an adapted version of the Q statistic (Q_A_) [34].

#### Sensitivity analyses

Sample overlap between exposure and outcome GWAS can bias MR estimates towards the confounded observational estimate (inflated type 1 error) in the presence of weak instrument bias in a manner proportional to the degree of overlap [37]. There was sample overlap across our MR analyses as the adiposity, ^1^H-NMR metabolite and EC GWAS all included participants from UK Biobank. Given this, we conducted sensitivity analyses to evaluate the influence of sample overlap in our MR analyses (Supplementary Methods).

### Examining off-target effects using PhenoScanner (Part V)

Due to the shared genetic architecture and pleiotropic nature of metabolite instruments [38–40], it is possible for variants acting as proxies for metabolites to exert off-target or pleiotropic effects on EC through other biological mechanisms. As such, intermediate associations may reflect common, but unmeasured, biological underpinnings rather than more obvious hypothesised pathway effects. To investigate this, we assessed whether genetic instruments for adiposity- and EC-associated, (and apparently intermediate) metabolites were associated with other traits (*p* < 1E-10) in a manner different to that expected by chance given documented genotype/metabolite/trait associations. Using PhenoScanner [41, 42], we assessed the co-association of adiposity- and EC-associated metabolites with other phenotypes and outcomes before performing the same procedure over 100 iterations, but for randomly selected metabolites taken from the 244 metabolites not found to underlie the association between adiposity and EC risk. The overall profile of these redraws were compared with that from the adiposity- and EC-associated instruments, i.e. counts of detectable associations with trait domains for adiposity- and EC-associated instruments versus SNPs associated with random metabolites.

## Results

### Population and data overview

The observational analyses within UK Biobank included up to 1,005 female participants who had a diagnosis of incident EC and up to 215,339 controls (Table 1). The cases and controls had a mean (SD) age of 59.63 (6.43) years and 55.83 (8.02) years, respectively. Mean BMI was higher among cases than controls (at 30.33 kg/m^2^ (7.07) and 27.06 kg/m^2^ (5.17), respectively). Mean WHR was higher among cases than controls (at 0.84 (0.07) and 0.82 (0.07), respectively). 3.66% of participants reported having a diagnosis of diabetes, and this was more common among cases than controls (8.99% and 2.64%, respectively).

**Table 1:** Baseline characteristics of UK biobank participants included in our study

	*N *(Cases/Control)	Endometrial cancer Cases (*N* = 1,005)	Controls (*N* = 215,339)
Continuous variables			
Age at assessment (year)	1005/215,339	59.63 (6.43)	55.83 (8.02)
Body mass index (kg/m^2^), mean (SD)	979/211,756	30.33 (7.07)	27.06 (5.17)
Waist to Hip Ratio, mean (SD)	1000/214,835	0.84 (0.07)	0.82 (0.07)
Age at first birth (year)	632/145,456	24.54 (4.23)	25.38 (4.64)
Age at last birth (year)	632/145,149	29.32 (4.55)	30.35 (4.90)
Menarche age (year)	971/208,424	12.64 (1.55)	12.98 (1.62)
Categorical variables			
Smoking	997/214,285		
Never		643 (64.49%)	129,857 (60.60%)
Previous		303 (30.39%)	65,775 (30.70%)
Current		51 (5%)	18,653 (8.70%)
Alcohol intake	1002/214,865		
Daily or almost daily		143 (14.27%)	34,152 (15.89%)
3–4 times a week		160 (15.97%)	44,464 (20.69%)
Once or twice a week		252 (25.15%)	55,476 (25.82%)
1–3 times a month		139 (13.87%)	28,241 (13.14%)
Special occasions only		193 (19.26%)	32,134 (14.96%)
Never		115 (11.48%)	20,398 (9.49%)
Diabetes	1001/214,512		
Yes		90 (8.99%)	7807 (2.64%)
No		911 (91%)	206,705 (96.36%)
Education	774/177,857		
College/University degree		304 (39.28%)	68,385 (38.45%)
A levels/AS levels or equivalent		106 (13.70%)	25,756 (14.48%)
O levels/GCSEs or equivalent		215 (27.78%)	49,943 (28.08%)
CSEs or equivalent		49 (6.33%)	11,924 (6.70%)
NVQ or HND or HNC or equivalent		33 (4.26%)	9676 (5.44%)
Other professional qualifications		67 (8.66%)	12,173 (6.84%)
Physical activity (No. of days/week of vigorous physical activity)	923/202,664		
0 days		394 (42.69%)	80,448 (39.70%)
1 days		156 (16.90%)	29,567 (14.59%)
2 days		148 (16.03%)	32,675 (16.12%)
3 days		106 (11.48%)	28,260 (13.94%)
4 days		46 (4.98%)	12,212 (6.02%)
5 days		36 (3.9%)	10,652 (5.26%)
6 days		12 (1.3%)	2676 (1.32%)
7 days		25 (2.71%)	6714 (3.31%)
Had Menopause	1002/214,649		
Yes		822 (82.04%)	126,379 (58.88%)
No		180 (17.96%)	88,270 (41.12%)
Ever used HRT	999/214,185		
Yes		382 (38.24%)	78,769 (36.78%)
No		617 (61.76%)	135,416 (63.22%)

### Association between adiposity measures and endometrial cancer (Part I)

**Fig. 2:**
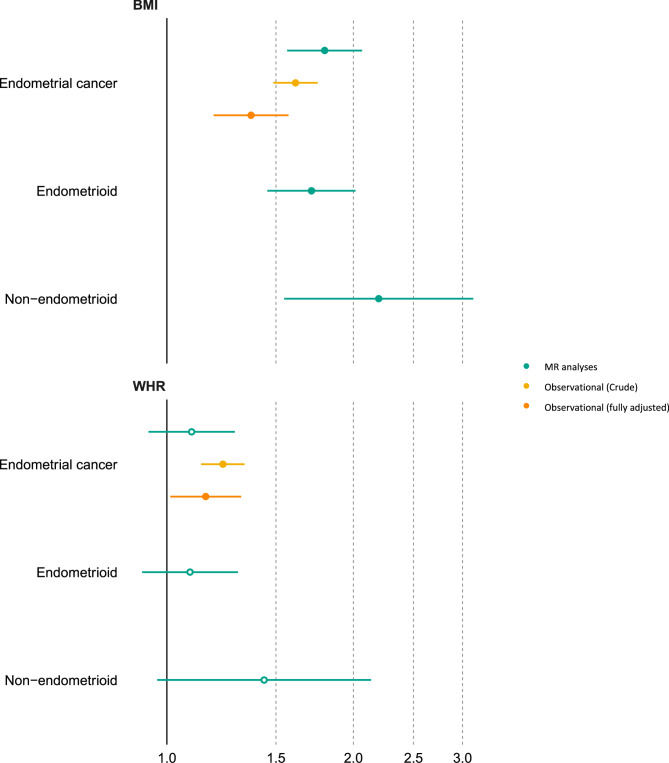
Mendelian randomization and observational estimates of the effect of BMI and WHR on endometrial cancer. The forest plot shows the estimates of the association between BMI and WHR with overall, endometrioid and non-endometrioid endometrial cancer from MR analyses (IVW method) using summary data from the Endometrial Cancer Association Consortium (ECAC) (*n*=12,906 endometrial cancer cases and 108,979 controls) and conventional observational analyses using individual level data from UK Biobank. Symbols represent point estimates from individual analyses. Horizontal lines represent 95% confidence intervals. Close circles represent p<0.05

In observational analyses, higher BMI (per SD) was associated with 1.61 (95% CI = 1.49, 1.75) times higher odds of overall EC; these results were consistent when adjusting for all covariables (OR = 1.37; 95% CI = 1.19, 1.57) (Fig. 2; Table 2). This was supported by univariable MR analysis, which found that higher BMI (per SD) was associated with 1.80 (95% confidence interval (CI) = 1.56, 2.07) times higher odds of overall EC using the IVW-MRE model (Fig. 2; Supplementary Table 5). Data on EC subtypes- endometrioid and non-endometrioid- were only available for MR analyses. Associations between BMI with endometrioid (OR = 1.71; 95% CI = 1.45, 2.02) and non-endometrioid cancer (OR = 2.20; 95% CI = 1.55, 3.12) was similar in magnitude to the overall EC MR analyses (Supplementary Table 5). Results from MR analyses were consistent across sensitivity analyses using methods that consider potential genetic pleiotropy and when using instruments from non-overlapping samples (Supplementary Table 5).

**Table 2:** Observational estimates of the association between adiposity measures and endometrial cancer.

Exposure	Outcome	Adjusted for age and centre		Fully adjusted	
		OR (95%CI)	*p*	OR (95%CI)	*p*
BMI	Incident EC	1.61 (1.49, 1.75)	7.63 × 10^−30^	1.37 (1.19, 1.57)	1.07 × 10^−5^
WHR	Incident EC	1.23 (1.14, 1.34)	4.30 × 10^−07^	1.15 (1.01, 1.32)	0.03

*BMI* Body mass index, *WHR* Waist hip ratio, *EC* Endometrial cancer, *OR* Odds ratio, *CI* Confidence interval, *p* p value

For WHR, observational analyses showed evidence that higher WHR (per SD) was associated with 1.23 (95% CI = 1.13, 1.34) times higher odds of overall EC; these results were consistent when adjusting for all covariables (OR = 1.15; 95% CI = 1.01, 1.32) (Fig. 2; Table 2). Results from MR analyses of WHR with overall EC (OR = 1.10; 95% CI = 0.93, 1.29), endometroid EC (OR = 1.09; 95% CI = 0.91, 1.30) and non-endometrioid EC (OR = 1.43; 95% CI = 0.96, 2.12) were not consistent with the observational analyses (Fig. 2; Supplementary Table 5). Given this, WHR was not taken forward for subsequent analyses.

### Association between adiposity measures and metabolites (Part II)

**Fig. 3:**
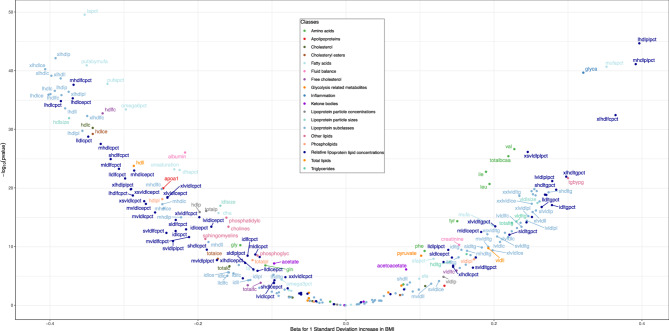
Mendelian randomization estimates of the effect of BMI on metabolites. The volcano plot shows the estimates of the effect of BMI on circulating metabolites from Mendelian randomization analyses. Metabolites associated with BMI at p<0.05 after correcting for multiple testing using Bonferroni correction are labelled

We identified 165 metabolites that were consistently associated with higher BMI (per SD) in observational and MR analyses (Fig. 3; Supplementary Tables 6 and 7; Supplementary Figs. 1 and 2). BMI had a broad effect on the metabolomic profile, with associations across many metabolite classes including numerous lipid metabolites and their ratios such as total cholesterol, total lipids, triglycerides and cholesteryl esters in high density lipoprotein (HDL) and very low-density lipoprotein (VLDL). For example, higher BMI led to 0.40 SD (95% CI = 0.34, 0.46) and 0.39 SD (95% CI = 0.38, 0.39) lower levels of total cholesterol in very large HDL in MR and observational analyses, respectively. Similarly, higher BMI led to 0.23 SD (95% CI = 0.19, 0.27) and 0.26 SD (95% CI = 0.25, 0.27) higher levels of valine in MR and observational analyses, respectively (Supplementary Table 6). Results from MR sensitivity analyses using instruments from non-overlapping samples were consistent (*r* = 0.94) (Supplementary Table 6). Given this, all 165 BMI-associated metabolites were taken forward for subsequent analysis with EC.

### Association between BMI-associated metabolites and endometrial cancer (Part III)

**Fig. 4:**
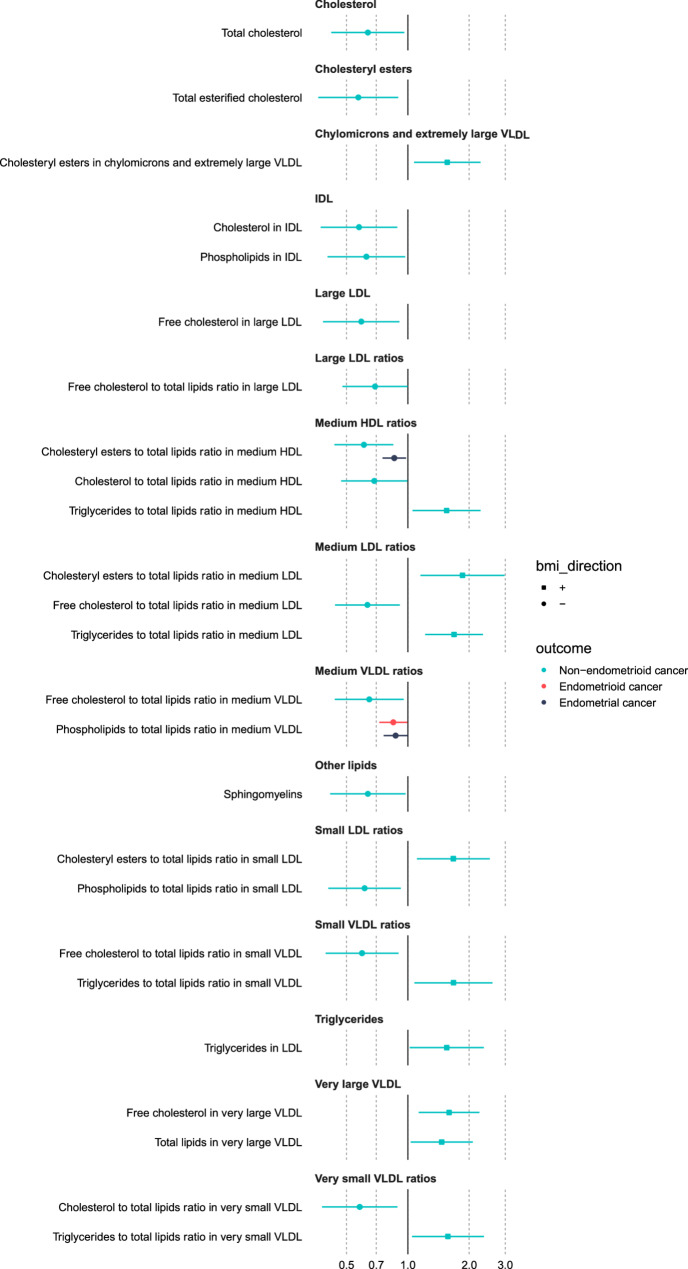
Estimates of the causal effect of the BMI-associated metabolites on endometrial cancer risk. The forest plot shows the estimate of the causal effect of the BMI-associated metabolites and overall, endometrioid and non-endometrioid endometrial cancer based on two-sample MR (IVW method) from female-specific analyses. Only metabolites associated with endometrial cancer with a p<0.05 are shown in this figure. Symbols represent point estimates from individual analyses. Horizontal lines represent 95% confidence intervals. Squares represent metabolites that are positively associated with BMI and circles represent metabolites that are negatively associated with BMI

Of the 165 metabolites associated with BMI in observational and MR analyses, 25 metabolites (representing 27 adiposity-metabolite-EC associations) were associated in MR analyses with EC in a direction (either increasing or decreasing risk) that was consistent with an intermediate role in the association between BMI and EC. This included 2 metabolites with overall EC, 1 metabolite with endometrioid EC, and 24 metabolites with non-endometrioid EC (Fig. 4 and Supplementary Fig. 3). These findings were broadly consistent across sensitivity analyses using instruments from non-overlapping samples (Supplementary Table 8). Results from observational analyses, which were only possible for overall EC, were consistent with results from MR analyses (Supplementary Table 9).

In MR analyses, of the 25 BMI-associated metabolites, 2 metabolites were associated with two EC outcomes: phospholipids to total lipids ratio in medium VLDL was associated with overall (OR = 0.87; 95% CI = 0.75, 1.00) and endometrioid EC (OR = 0.85, 95% CI = 0.72, 0.99) and cholesteryl esters to total lipids ratio in medium HDL was associated with overall (OR = 0.86, 95% CI = 0.75, 0.98) and non-endometrioid EC (OR = 0.61, 95% CI = 0.44, 0.85). The remaining 23 metabolites - mainly of the lipoprotein subclasses - were associated with just one EC outcome, which was predominantly non-endometrioid cancer. For example, the largest positive and negative effects were observed for the association between cholesteryl esters to total lipids ratio in medium LDL (OR = 1.85; 95% CI = 1.15, 2.98) and total esterified cholesterol (OR = 0.57; 95% CI = 0.36, 0.90) and non-endometrioid cancer(Fig. 4).

### Examining the intermediate role of metabolites in the effect of adiposity on EC: Multivariable MR analyses (Part IV)

**Fig. 5:**
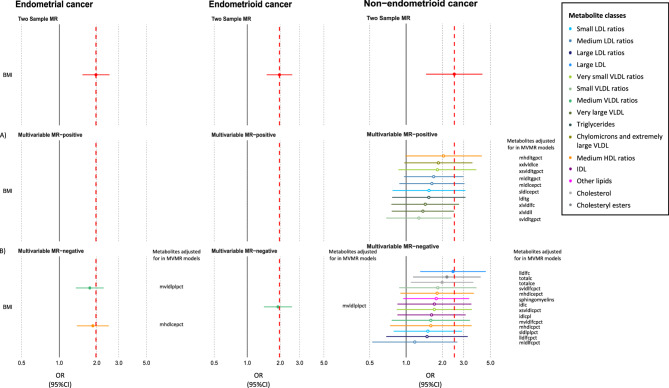
Multivariable mendelian randomization estimates of the direct effect of BMI on endometrial cancer after adjustment for various BMI-associated metabolites. The forest plot shows the estimate of the direct causal effect of BMI on overall, endometrioid and non-endometrioid endometrial cancer based on multivariable MR analyses after adjustment for various metabolites that were associated with A) increased association with both BMI and endometrial cancer or B) decreased association with both BMI and endometrial cancer. Symbols represent point estimates of the direct effect of BMI on endometrial cancer after adjustment for various BMI-associated metabolites separately from multivariable MR analyses. Dotted red line represents the estimates of the indirect effect of BMI on endometrial cancer obtained from univariable IVW analyses

We used MVMR to investigate whether the 25 BMI-associated metabolites were potential intermediates of the effect of BMI on EC risk. In MVMR, there was little evidence that the association of BMI with overall and endometrioid EC was strongly attenuated following adjustment for most metabolites (Fig. 5**)**. For non-endometrioid EC, the association of BMI was attenuated following adjustment for various metabolites (Fig. 5); for example, the univariable MR OR for non-endometrioid EC per 1 SD higher BMI was 2.51 (95%CI = 1.47, 4.29), whereas the MVMR OR for non-endometrioid EC per 1 SD increase in BMI was 1.18 (95%CI = 0.53, 2.66) after adjusting for free cholesterol to total lipids ratio in medium LDL. The conditional F statistics for BMI and metabolites instruments are presented in Supplementary Table 10.

### Examining off-target effects

**Fig. 6:**
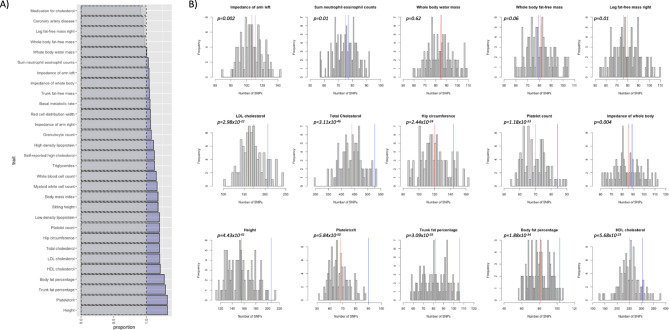
Traits associated with genetic instruments for metabolites underlying effect of BMI on endometrial cancer. **A** The blue bars represent the proportion of genetic instruments associated with the top 30 traits identified in PhenoScanner for metabolites underlying the effect of BMI on endometrial cancer relative to the negative control (grey bars). The negative control was obtained by iteratively (*N*=100) looking up traits associated with instruments for 25 randomly selected metabolites not underl ying the association between BMI and endometrial cancer. **B** the histograms represent the frequency of the number of SNPs associated with trait from the negative control analysis. The red line represents the mean of the number of SNPs associated with each trait for the negative control and the blue line represents the number of SNPs associated with the metabolites underlying the effect of BMI on endometrial cancer risk associated with each trait

Given the highly pleiotropic nature of the metabolite instruments, it is possible that the instruments might be proxying other common, but unmeasured, biological pathways rather than having a direct effect through the metabolites. Using a commonly used catalogue of genetic association results (PhenoScanner [41, 42]), we conducted a phenome scan for the genetic instruments proxying metabolites potentially underlying the adiposity-EC relationship and compared the traits association profiles with those of randomly selected metabolites. We identified a larger than expected proportion of instruments associated with several lipid traits including HDL, LDL, triglycerides and total cholesterol, as well as anthropometric (height and adiposity) and blood cell traits for the metabolites underlying the adiposity-EC relationship compared to the randomly selected metabolites. This suggests that the metabolite instruments could be proxying for or are a response read-out of other potentially shared biological pathways (Fig. 6).

## Discussion

We found consistent evidence from observational and MR analyses that higher BMI increased risk of overall EC and EC stratified by histological subtypes, but did not find similar consistency for WHR. We further identified 165 metabolites associated with higher BMI in observational and MR analyses, 25 of which were also associated with EC outcomes in a manner consistent with potential intermediate roles in the BMI-EC association. There was evidence from both two-step and multivariable MR analyses that the effect of BMI on EC was mediated strongly by several lipid metabolites; however, further bioinformatic analysis highlighted numerous potential pleiotropic and shared mechanisms that could potentially explain associations and patterns of intermediate effect.

Adiposity is a well-recognised risk factor for EC [43–45] and our results are consistent with findings from recent MR studies where genetically elevated BMI, but not WHR, was found to be causally linked to EC [6, 8, 46, 47]. It is well-established in the literature that obesity is associated with both endometrioid and non-endometrioid EC, with stronger associations of an effect on endometrioid EC, as compared to non-endometrioid EC [6, 8, 46, 48–54]. Interestingly, the effect of BMI on non-endometrioid EC was slightly greater than endometrioid EC in our study. Little is known about the etiology of non-endometrioid EC and although we see some evidence of metabolites as intermediates in the association with BMI, the association with BMI warrants further investigation to understand whether these reflect mechanisms or markers of biological processes.

Previous work has shown that adiposity leads to perturbations in systemic metabolism which may have an impact on EC [16, 55]. In both MR and observational analyses, BMI was associated with a wide array of metabolites, many of which have been previously reported [15, 36, 56, 57]. However, several of the associations we observed were inconsistent with those previously reported. This included the associations of BMI with LDL cholesterol, total cholesterol, sphingomyelin, and fatty acids [15]. It has been suggested that the prevalence of statin users in UK Biobank (22% of men and 12% of women) may impact metabolomic analyses [56] which may explain some of these differences.

Our two-step and multivariable MR analyses suggested that several lipid metabolites - the majority of which were metabolite ratios - could be potential intermediates between BMI and non-endometrioid EC risk. We found associations between triglycerides (including their ratios to LDLs of varying sizes) with EC risk, supporting evidence that triglycerides may affect EC development through peroxisome proliferator-activated receptors (PPAR) deregulation [58]. While our results for triglycerides are consistent with several observational studies [58–61], they are inconsistent with a recent MR analysis which found little evidence of a causal link [62]. We also identified other potential intermediates including components of the cholesterol metabolism pathway, which are known to influence the onset and progression of cancer [63], including endometrial cancer [64, 65]. Specifcally, higher cholesteryl esters to total lipids ratio in small and medium LDL were positively associated with both BMI and non-endometrioid EC risk while free cholesterol to total lipids ratio in medium and large LDL were inversely associated with BMI and reduced non-endometrioid EC risk. These metabolite ratios associations may reflect flux through biochemical pathwys, highlighting the need for further work to examine pathway effects which may better reflect the complex nature of metabolites.

Major challenges persist for identifying instruments to proxy circulating metabolites due to the complex genetic architecture of blood metabolites [38–40]. The high correlational structure of many metabolites means instruments for metabolites are often associated with other metabolites [40, 66]. While, the high degree of pleiotropy (or basic biological overlap) for metabolite instruments with other modifiable risk factors and disease endpoints, makes determining the exact molecular mechanism by which they impact the outcome difficult. Indeed, we identified other shared pathways with our metabolite instruments, including height and several adiposity and blood cell traits, that could influence associations with EC. This suggests that when compared with a (hypothetically null) set of randomly selected metabolites, instruments for implicated metabolites could be flagging biological pathways that may be driving causal associations. However, it is difficult to determine whether the biological pathways highlighted are due to vertical pleiotropy (i.e., the mechanism by which the SNP influences the outcome is via metabolites) or horizontal pleiotropy (i.e., the SNP could influence the outcome through a pathway independent of metabolites). Our approach to the examination and characterisation of signal metabolites is conceptually similar to a cis-versus-trans MR analysis of proteins that looks to clarify inference from MR methods through prior information available on the instruments in question. Using this approach, a recent study [67] demonstrated that the observed relationship between small HDL particle count and sepsis were - in part - driven by potential confounding between interleukin 6 (IL-6) and HDL, where IL-6 signalling is the true mechanism.

### Strengths and limitations

This work adds to the body of evidence suggesting a causal relationship between adiposity and adiposity-associated metabolites with overall, endometrioid, and non-endometrioid EC risk. We focussed predominantly on lipid-based metabolites identified via ^1^H-NMR metabolomics rather than mass spectrometry, which is not yet available at sufficient scale. Mass spectrometry-based metabolomic analyses offers a broader representation of metabolites beyond lipid subclasses and there is a growing body of evidence that metabolites detected by mass spectrometry are altered in EC patients [12, 68]. Future metabolomic studies using mass spectrometry should be conducted to comprehensively evaluate the role of metabolites as intermediates between adiposity and EC.

Given the common shared genetic architecture, high correlation structure, and shared biology of metabolites, it is likely that a perturbation in any one metabolite does not happen in isolation. This is exemplified in recent work which has shown that the variance explained by a metabolite’s instrument is often greater for another [39, 66, 69]. Here, we use a naïve approach to instrumentation in a hypothesis generating analysis; however, given many metabolite instruments include only a handful of SNPs, another limitation of our study is that statistical methods aiming to measure the potential effects of pleiotropy (e.g., MR-Egger) do not always lead to meaningful results. This also applies to techniques designed to evaluate the effect of multiple correlated exposures (e.g., MVMR).

In two-sample univariable MR analyses, sample overlap can bias estimates towards the confounded observational estimate (inflated type 1 error) in the presence of weak instruments in a manner proportional to the degree of overlap [37]. A limitation of our main MR analyses is that we likely have 100% overlap between the adiposity and metabolites GWAS and up to a maximum 5% overlap for both of these with the overall EC GWAS. However, given bias due to sample overlap is negligible in the presence of strong instruments [70] and that the results across sensitivity analyses using non-overlapping samples were generally consistent with the main analyses, it is unlikely that estimates from our analyses are meaningfully impacted by such bias.

Another limitation is the unrepresentative nature of UK Biobank (initial response rate ~ 5%) [71] and the potential for selection bias. Given the age of the UK Biobank participants, the prevalence of statin use is high (~ 16% in UK Biobank vs. ~ 11% in the general UK adult population in 2014 [72]). For our MR analyses, we used summary-level data from the ^1^H-NMR metabolite GWAS conducted using data from UK Biobank. This approach limits the capacity to fully explore the effects of other factors such as age and medication use (including statin use and HRT), which may influence the association between adiposity traits, metabolites and EC. Our analyses were limited to women of European ancestries; thus, these findings may not apply to individuals of other ancestries. Furthermore, given that we used the same ^1^H-NMR data from UK Biobank for both our MR and observational analyses examining the associations between adiposity traits and metabolites, these analyses cannot be considered independent. Replication of this study in other large cohort studies and in other ancestries will allow a more robust characterisation of the metabolic profile associated with adiposity and the subsequent impact on EC.

Lastly, our study primarily examined the effect of excess adiposity on endometrial development. Robust evidence supports the association between weight loss interventions—including bariatric surgery—and reduced endometrial cancer incidence. While it is well-established that excess adiposity increases the risk of recurrence and mortality among endometrial cancer survivors [73, 74], the effect of bariatric surgery on endometrial cancer recurrence remains unclear and warrants further investigation.

## Conclusions

Our study suggests that higher BMI causes a higher risk of overall and all histological subtypes of EC and variation in numerous circulating metabolites. Several of these metabolites showed relationships consistent with an intermediate role in the effect of BMI on non-endometrioid EC; however, these relationships may be a result of other potential shared mechanisms.

## Supplementary Information

Supplementary Material 1.

Supplementary Material 2.

Supplementary Material 3.

Supplementary Material 4.

Supplementary Material 5.

## Data Availability

Data from UK Biobank were accessed via application number 16391 and 30418. Data for adiposity measures and endometrial cancer outcome was extracted on the 08/07/2021 and the 1H-NMR metabolite data was extracted on the 08/02/2023. The individual level data used in this work is not publicly available and can only be obtained from UK Biobank with an approved application. All analyses were performed using R version 3.5.333. Multivariable linear and logistic regression analyses were performed using the lm and glm functions, respectively, in R. Univariable MR analyses were performed using TwoSampleMR26 (version 0.4.22).; multivariable MR analyses were performed using MVMR)32 (version 0.3) R package; forest plots were created using ggforestplot (version 0.1)(74) R package; circos plots were created using EpiViz [75–78] (version 0.1) R package. All publicly available data, code, and results used in this work are available on https://github.com/mattlee821/adiposity_metabolites_endometrial_cancer. This includes all exposure data used in all MR analyses. The full summary statistics for BMI and WHR are available from the GIANT consortium (https://portals.broadinstitute.org/collaboration/giant/index.php/GIANT_consortium_data_files) and Zenodo (https://zenodo.org/record/1251813#.Yk7O25PMIUE), data files 4, 5, 7, and 8. The full summary statistics for the endometrial cancer GWAS are available from the OpenGWAS database (https://gwas.mrcieu.ac.uk/datasets/); IDs for endometrial cancer GWAS: ebi-a-GCST006464, ebi-a-GCST006465, and ebi-a-GCST006466; This can be accessed via the TwoSampleMR (https://mrcieu.github.io/TwoSampleMR/) and ieugwasr (http://gwas-api.mrcieu.ac.uk/) R packages or directly from OpenGWAS in GWAS-VCF format50. The full summary statistics for the 1H-NMR metabolites will be made available at the University of Bristol data repository.
