# Supplementary material for: Adiposity, metabolites and endometrial cancer risk: inference from combinations of Mendelian randomization and observational analyses
Source: BMC Cancer. 2025 Oct 21;25:1619. doi: 10.1186/s12885-025-14756-y (PMC12539199; doi:10.1186/s12885-025-14756-y)
Supplement: Supplementary file 2 — Supplementary Material 2. [file 12885_2025_14756_MOESM2_ESM.docx]

#### **Supplementary Methods**

#### **Metabolite GWAS in UK Biobank and selection of metabolite instruments**

A random subset of non-fasting plasma samples, consisting of 275,000 UK Biobank participants and 17,000 repeat-visit samples (around 15,500 of these have both a baseline and repeat assessment), were measured using targeted high-throughput ^1^H-Nuclear magnetic resonance (^1^H-NMR) metabolomics (Nightingale Health Ltd; biomarker quantification version 2020)^1,2^. Genotype data was available for 488,377 individuals, of which 49,979 were genotyped using the UK BiLEVE array and 438,398 using the UK Biobank axiom array. Pre-imputation QC, phasing and imputation have been described previously^3^. Genotype imputation was performed using IMPUTE2 algorithms^4^ to a reference set combining the UK 10K haplotype and HRC reference panels^5^. Post-imputation QC was performed as described in the “UK Biobank Genetic Data: MRC-IEU Quality Control” documentation^6,7^. For the GWAS, we restricted the samples to individuals of European ancestry as defined by the largest cluster in an in-house k-means clustering of genetic ancestry data (K=4) after standard exclusions including withdrawn consent, mismatch between genetic and reported sex and putative sex chromosome aneuploidy^6–8^ (**Supplementary Table 3**). Metabolite measures were inverse rank normal transformed prior to genome-wide analysis and units therefore represent a normalised SD. Population structure was modelled using 143,006 directly genotyped SNPs (MAF > 0.01; genotyping rate > 0.015; Hardy-Weinberg equilibrium p-value < 0.0001 and LD pruning to an r2 threshold of 0.1 using PLINK(v2.00)). Genotype array and fasting time were adjusted for in the model.

**Sensitivity analyses**

Sample overlap between exposure and outcome GWAS can bias MR estimates towards the confounded observational estimate (inflated type 1 error) in the presence of weak instrument bias in a manner proportional to the degree of overlap^9^. This bias can be inflated by ”Winner’s curse”, in which weights for genetic instruments are derived from discovery samples that overlap with outcome samples. There was sample overlap across our MR analyses as the adiposity, ^1^H-NMR metabolite and EC GWAS all included participants from UK Biobank. Given the random selection of samples for metabolomics analysis and the inclusion of almost all samples within the adiposity GWAS it is not possible to precisely quantify the degree of sample overlap but we make the assumption of 100% sample overlap between the adiposity and ^1^H-NMR metabolite GWAS. There was also approximately 5% overlap for both the adiposity and ^1^H-NMR metabolite GWAS with the overall EC GWAS. Given this, we conducted sensitivity analyses to evaluate the influence of sample overlap in our MR analyses. First, for analyses examining the association between adiposity traits with endometrial cancer risk (**Figure 1, Part I**), we re-performed MR analyses using alternative GWAS data for BMI (n=171,977) and WHR (N=118,004) where there was no sample overlap^10,11^ with either the EC GWAS conducted by ECAC^12^ or an alternative EC GWAS conducted by ECAC which excluded UK Biobank participants (personal correspondence) (N for overall EC=12,270 cases and 46,126 controls; N for endometrioid EC=8,758 cases and 46,126 controls and N for non-endometrioid EC = 1,230 cases and 35,447 controls)^12^. Second, for analyses examining the association between adiposity traits with ^1^H-NMR metabolites (**Figure 1, Part II**), we re-performed MR analyses using alternative GWAS data for BMI where there was no sample overlap^10,11^. Third, for analyses examining the association between ^1^H-NMR metabolites and endometrial cancer risk (**Figure 1, Part III**), we re-performed MR analyses using the alternative GWAS for EC which had excluded UK Biobank participants^12^ (**Supplementary Table 3**).

**References**

1. Soininen, P. *et al.* High-throughput serum NMR metabonomics for cost-effective holistic studies on systemic metabolism. *Analyst* (2009) doi:10.1039/b910205a.

2. Würtz, P. *et al.* Quantitative serum nuclear magnetic resonance metabolomics in large-scale epidemiology: a primer on-omic technologies. *Am J Epidemiol* **186**, 1084–1096 (2017).

3. Bycroft, C. *et al.* The UK Biobank resource with deep phenotyping and genomic data. *Nature* **562**, (2018).

4. Howie, B., Marchini, J. & Stephens, M. Genotype Imputation with Thousands of Genomes. *G3&amp;#58; Genes|Genomes|Genetics* **1**, 457–470 (2011).

5. Huang, J. *et al.* Improved imputation of low-frequency and rare variants using the UK10K haplotype reference panel. *Nat Commun* **6**, 8111 (2015).

6. Mitchell, R, Hemani, G, Dudding, T, Paternoster, L. UK Biobank Genetic Data: MRC-IEU Quality Control, Version 1. (2017) doi:https://doi.org/10.5523/bris.3074krb6t2frj29yh2b03x3wxj.

7. Mitchell R, Hemani G, Dudding T, Corbin L, Harrison S, P. L. UK Biobank Genetic Data: MRC-IEU Quality Control, version 2. https://doi.org/10.5523/bris.1ovaau5sxunp2cv8rcy88688v (2019).

8. Anderson, C. A. *et al.* Data quality control in genetic case-control association studies. *Nat Protoc* (2010) doi:10.1038/nprot.2010.116.

9. Burgess, S., Davies, N. M. & Thompson, S. G. Bias due to participant overlap in two-sample Mendelian randomization. *Genet Epidemiol* **40**, (2016).

10. Locke, A. E. *et al.* Genetic studies of body mass index yield new insights for obesity biology. *Nature* **518**, 197–206 (2015).

11. Shungin, D. *et al.* New genetic loci link adipose and insulin biology to body fat distribution. *Nature* **518**, 187–196 (2015).

12. O’Mara, T. A. *et al.* Identification of nine new susceptibility loci for endometrial cancer. *Nat Commun* **9**, 3166 (2018).
